# PTEN and rapamycin inhibiting the growth of K562 cells through regulating mTOR signaling pathway

**DOI:** 10.1186/1756-9966-27-87

**Published:** 2008-12-30

**Authors:** Zhi Y Cheng, Xiao L Guo, Xiao Y Yang, Zhi Y Niu, Shi H Li, Su Y Wang, Hao Chen, Ling Pan

**Affiliations:** 1 Department of Hematology and Heibei Institute of Hematology, The Second Hospital of Hebei Medical University, 215 Heping Xi Road, Shijiazhuang,050000, PR China

## Abstract

**Objective:**

To investigate, *in vitro*, the regulatory effects of tumor-suppressing gene PTEN on mTOR (mammalian target of rapamycin) signaling pathway, the effects of transfected PTEN and rapamycin on the growth inhibition, and apoptosis induction for human leukemia cell line K562 cells.

**Methods:**

K562 cells were transfected with recombined adenovirus-PTEN vector containing green fluorescent protein (Ad-PTEN-GFP), followed by the treatment of the cells with or without rapamycin. The proliferation inhibition rate and apoptotic rate of these transfected and/or rapamycin treated K562 cells were measured by MTT assay and flow cytometry (FCM), the expression levels of PTEN-, mTOR-, cyclinD1- and P27^kip1^- mRNA were measured by real-time fluorescent relative-quantification reverse transcriptional PCR (FQ-PCR), the protein expression levels of PTEN, Akt, p-Akt were detected by western blotting.

**Results:**

The proliferation of K562 cells was inhibited by PTEN gene transfection with/without the treatment of rapamycin. The expression levels of PTEN- and P27^kip1^- mRNA were up-regulated, and the mTOR- and cyclinD1- mRNA were down-regulated in K562 cells after the cells transfected with wild type PTEN gene and treated with rapamycin.

**Conclusion:**

PTEN and rapamycin inhibited mTOR expression by acting as an upstream regulator of mTOR. Low dose rapamycin in combination with over-expressed PTEN might have synergistic effects on inhibiting the proliferation and promoting apoptosis of K562 cells.

## Background

PTEN (phosphatase and tensin homology deleted on chromosome ten) is a novel tumor suppressor gene, mapping to 10q23.3 and encoding a dual-specificity phosphatase with sequence similar to the cytoskeletal protein tensin [1,2]. It was identified as a candidate tumor suppressor gene based on the presence of gene deletion or inactivating mutations in human brain, breast, and prostate cancers and tumor cell lines [3-6]. PTEN protein can be inactivated by post-transcriptional gene silencing, post-transcriptional modifications and GpG island methylation [3-8].

mTOR (mammalian target of rapamycin) gene locates in the downstream of PI3K/Akt signal pathway, encoding a serine/threonine protein kinase which is considered as an oncoprotein. Studies showed that PI3K/Akt/mTOR and MAPK and FAK signaling pathways and their downstream partners were activated in leukemia, inducing the decontrolled proliferation and enhancing the invasiveness of leukemia cells [9,10].

PTEN inhibits the activation of the above mentioned pathways via its lipid phosphatase and protein phosphatase activities [8]. Rapamycin (RAPA), a triene macrolide antibiotic, has anti-mTOR activity by binding mTOR to interfere the interaction of mTOR with other target proteins in the signaling pathway. Targeting mTOR signaling pathway can be a new strategy for the treatment of leukemia [10-13].

Several studies showed that down-regulation of PTEN mRNA expression and protein synthesis, and PI3K/Akt/mTOR pathways were activated in the bcr/abl fusion protein positive K562 cells. However, the relationship between the high expression of PTEN and the inactivation of mTOR signal pathway and their effects on the proliferation of K562 cells were not clear. The present study was designed to transfect K562 cells with recombined adenovirus-PTEN vector containing green fluorescent protein (Ad-PTEN-GFP) or Ad-GFP vector followed by the treatment of the cells with or without rapamycin. The regulatory mechanism of PTEN and rapamycin on mTOR signaling pathway in K562 cells was then explored and the growth inhibition effect of this treatment on K562 cell was analyzed.

## Materials and methods

### Main reagents

Chemicals were purchased from Sigma-Aldrich and Qiangen Biotechnology (Qiangen, Beijing). Antibodies to PTEN, Akt, p-AKT (serine 473), GAPDH were purchased from Santa Cruz Biotechnology (Santa Cruz, CA). Secondary antibodies for western blot analysis (horseradish peroxidase-conjugated anti-mouse Ig) were purchased from Beijing Ding Guo biotechnology CO. LTD. Rapamycin (RAPA) was from North China Pharmaceutical Group Corporation. Other reagents were of analytical grade.

### Cell culture

Human embryonic kidney cell line 293A cells were cultured in the medium of HD DMEM, supplemented with 10% fetal bovine serum and used for adenovirus amplification. K562 cells, a cell line established from a chronic myelogenous leukemia patient in blast crisis, were cultured in RPMI1640 supplemented with 10% fetal bovine serum, glutamine (2%), penicillin (100 U/ml) and streptomycin (100 μg/ml) at 37°C in a humidified atmosphere with 5% CO_2_. Exponentially growing K562 cells were seeded into flat-bottomed tissue-culture plates at the density of 5 × 10^5 ^cells/ml and cultured overnight

### Cell transfection with Adenovirus Vectors

The first generation of recombinant adenovirus was generated by Jikai biological CO.LTD, Shanghai. The particle titers of the adenoviral stocks were typically 1 × 10E9 plaque-forming units per milliliter (pfu/mL). Adenovirus amplification was propagated in 293A cells for several times to obtain high-titer stocks, as determined by the plaque assay. Viral stocks of 1 × 10E9 pfu/mL were kept at -80°C. Adenovirus vectors expressing the transgene PTEN, containing green fluorescent protein (Ad-PTEN-GFP) or empty vectors (Ad-Vector) were used to transfect the K562 cells. At the condition when multiplicity of infection (MOI) was 200, K562 cells were co-cultured with Ad-PTEN-GFP or Ad-GFP at 37°C, in a humidified atmosphere with 5% CO_2_, for 2 h. The transfection efficiency was detected directly by FMC for the expression ratio of green fluorescent protein.

### Measurement of the effects of PTEN gene-transfection on K562 cells

From the first day to the seventh day after the transfection, cell growth inhibition rate of the transfected K562 cells from each group was measured by MTT method and FCM, the mRNA expression levels of PTEN, mTOR, cyclinD1, P27^kip1 ^and BCR/ABL fusion gene in the transfected K562 cells were detected by FQ-PCR and the protein levels of PTEN, Akt, p-Akt were measured by western blotting.

### Measurement of growth inhibition effect of rapamycin on K562 cells

Raparamcin (RAPA) was dissolved in DMSO and then added to the culture medium of the transfected K562 cells or control K562 cells at the final concentrations of 0, 1, 5, 10, 20, 50 and 100 nmol/L. The effects of rapamycin, together with PTEN transfection, on cell proliferation, apoptosis and cell cycle were detected by MTT assay and FCM when the K562 cells were treated with rapamycin for 12, 24, 48 or 72 hours. The mRNA expression levels of PTEN, mTOR, cyclinD1, P27^kip1 ^and BCR/ABL fusion gene in the rapamycin reated and PTEN transfected K562 cells were detected by FQ-PCR and the protein levels of PTEN, Akt, p-Akt by western blotting.

### Cell cycle distribution assay

For the cell cycle analysis, K562 cells (at least 1 × 10^5 ^cells) from each group were collected at each culture-ending point, washed and fixed with 70% ethanol, placed at 4°C overnight. Then, the cells were treated with RNA enzyme for 30 minutes before the addition of propidium iodide (PI), and then placed at 4°C for more than 15 minutes. Finally, the cell cycle distribution (G_0_/G_1 _phase, S, G_2_/M) and cell apoptosis rate was detected by FCM

### Measurement of the target genes by FQ-PCR

K562 cells were collected from each group, washed with PBS three times. Total RNA was extracted from the cells according to the RNeasy Mini Protocol (Qiangen Biothch, Beijing). Each 1 μg total RNA from each group of K562 cells was reversely transcribed in a 20 μL reaction mixture which contained 0.5 μg oligo (dT)_15 _as primer by SuperScript II reverse transcriptase (Promage). The resultant cDNA was subjected to FQPCR. PCR was carried out in a total 25 μl reaction system, with the SYBR Green I PCR Reagent kit (Qiangen Biothech, Beijing) and a Lightcycler fluoresencent quantitative amplification analyzer (Bio-Rad, Hercules, CA). The reaction condition was 94°C for 5 min, 94°C 45s and 60°C 1 min, for 30 cycles. The fluorescence signal of the first 3 to 15 cycles was settled as fluorescent background signal and the baseline was adjusted accordingly. Relative quantification of gene expression level was calculated by the following method: for each reaction to amplify the target gene and β-actin gene, the Ct value was determined. The ratio of of each target mRNA to β-actin mRNA was calculated by the formula 2^-ΔCt^. The specific primers' sequences for the target genes were shown in Table 1.

**Table 1:** Primer sequence for FQPCR

Primers	Sequence	Product size	Accession number from genebank
BCR/ABL	F : 5'- GGAGCTGCAGATGCTGACCAAC-3'	200 bp	AY789120
	R : 5'- TCAGACCCTGAGGCTCAAAGT-3'		
PTEN	F : 5'-ATACCAGGACCAGAGGAAACC-3'	101 bp	000314
	R : 5'-TTGTCATTATCCGCACGCTC-3'		
mTOR	F : 5'- GAGCCAGTGTTCCCTCCAT-3'	128 bp	NM_152756
	R : 5'- CAGTCAGCGGCCAGTCAT-3'		
CyclinD1	F : 5'-CGATGCCAACCTCCTCAACGAC-3'	143 bp	NM_053056
	R : 5'-CCAGCATCCAGGTGGCGACG-3		
P27^kip1^	F : 5'-ACGTGAGAGTGTCTAACGG-3'	138 bp	NM_004064
	R : 5'-AGTGCTTCTCCAAGTCCC-3'		
β-actin	F : 5'- CTGGCACCACACCTTCTACAAT -3'	382 bp	NM_001101
	R : 5'- AATGTCACGCACGATTTCCCGC -3'		

### Measurement of the target proteins by western blotting

Western blot analysis was performed for detecting the expression level of specific protein in freshly washed K562 cells from each group. The protein concentrations of the cell lysate were measured by coomassie brilliant blue kit (Kang Chen Biotechnology, Beijing, China). Each 50 μg of the cell lysate was loaded onto a 10% SDS-PAGE gel, and run at 100 volts for 2 h. Cell proteins were transferred to nitrocellulose membrane, blocked with 5% milk in TBS (Tris-buffered saline with 0.1% Tween 20) for 1 h, then, washed and incubated with the primary antibody for 1 h at room temperature or overnight at 4°C. The blots were washed and incubated with the horseradish peroxidase-conjugated secondary antibody, and developed with a chemiluminescent substrate, ECL Plus. Following development of the primary antibodies, the bounded immunoglobulins were removed from the membranes by washing twice, each time for 15 minutes at room temperature in Restore Western blot Stripping Buffer. An autoradiograph was obtained, and protein levels were measured using a Fluors scanner and Quantity One software for analysis (Bio-Rad).

### Statistical analysis

tatistical analysis was done by Student's *t*-test and Spearman's test. Data was shown by mean ± SD of three independent experiments. The results from each group were compared with that of the corresponding control. *P *< 0.05 was considered as statistically significance.

## Results

### Anti-proliferation effect of PTEN transfection in K562 cells

**Figure 1:**
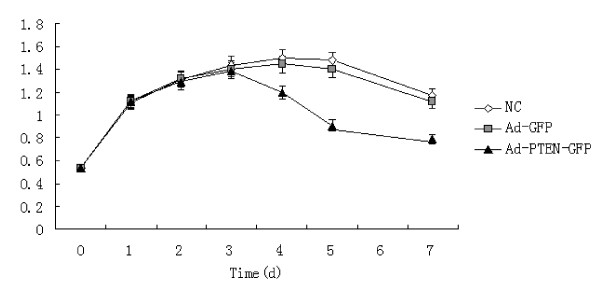
**Anti-proliferation effect of PTEN geng transfecion in K562 cells NC: **controlled K562 cells; Ad-GFP: Ad-GFP tranfected K562 cells; Ad-PTEN-GFP: Ad-PTEN-GFP tranfected K562 cell

Our preliminary experiment and several published papers showed that K562 cells expressed PTEN mRNA and protein at very low levels [8]. Therefore, K562 cells were transfected with Ad-PTEN-GFP or Ad-GFP in the present study. After the transfection, the difference in cell growth inhibition rate between K562 cells from each group was not statistically significant in the first 3 days. The maximal growth inhibition rate of 35.2% in Ad-PTEN-GFP transfected K562 cells was detected at the fifth day and that was statistically significant higher than that in Ad-GFP transfected K562 cells and that in control K562 cells (*P *< 0.01 Fig 1).

### PTEN transfection regulated the expression of specific genes and proteins in K562 cells

**Figure 2:**
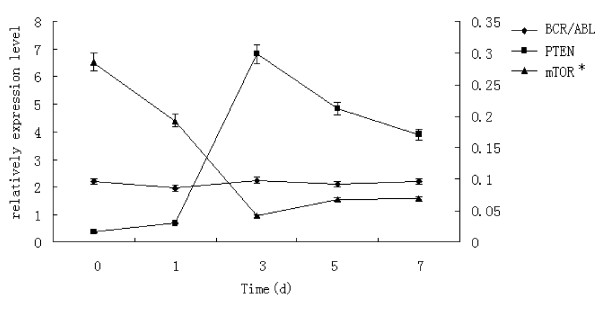
**The expression levels of BCR/ABL, mTOR and PTEN mRNA in K562 cells transfected with PTEN gene (MOI = 200)**. *mTOR to right ordinate When the expression of PTEN mRNA increased, the expression of mTOR mRNA decreased in K562 cells after the cells transfected wild-type PTEN, but BCR/ABL fusion gene did not change significantly.

**Figure 3:**
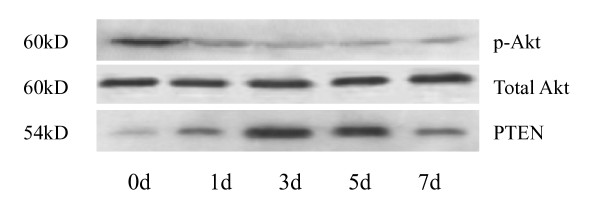
**Akt, p-Akt and PTEN protein expression levels in K562 cells transfected with PTEN for different time point**. The expression of p-Akt- protein in K562 cells decreased gradually following Ad-GFP-PTEN transfection, and the lowest level appeared on the third day of the transfection. PTEN protein increased gradually after Ad-GFP-PTEN transfection, and the top level was detected on the third day of the transfection. However, there was no obvious change in total Akt expression.

After transfection of Ad-PTEN-GFP to K562 cells, the expression level of PTEN mRNA increased gradually, reached the top level on the third day and then decreased gradually in the following three days, while the expression level of mTOR mRNA decreased to the lowest level on the third day and recovered gradually in the following three days. However, the expression level of BCR/ABL fusion gene did not change significantly (*P *> 0.05) (Fig 2). The expression level of PTEN mRNA had negative correlation with that of mTOR mRNA (r = -0.956, *P *< 0.05). The result also showed that the expression level of PTEN protein increased gradually after Ad-GFP-PTEN transfection, and the top level was detected on the third day of the transfection. The expression level of p-Akt but not total Akt decreased in PTEN transfected K562 cells, the lowest level appeared on the third day (Fig 3).

### PTEN transfection changed the cell cycle distribution in K562 cells

**Figure 4:**
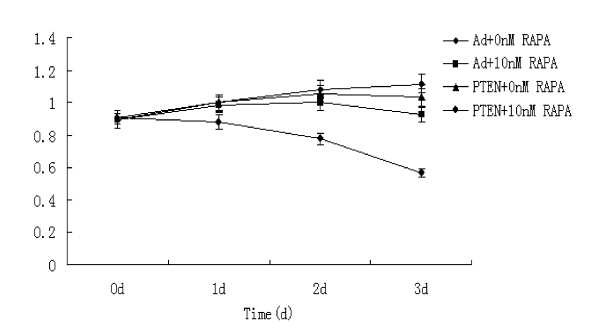
**The cell cycle distribution of K562 cells transfected with Ad-PTEN-GFP or Ad-GFP and treated with or without 10 nM RAPA detected by flow cytometry**. A: transfected with Ad-GFP; B: transfected with Ad-GFP and treated with 10 nmol/L RAPA; C: transfected with Ad-PTEN-GFP; D: transfected with Ad- PTEN-GFP and treated with 10 nmol/L RAPA. After treated with 10 nmol/L RAPA for three days, the apoptosis rate of K562 cells was 31.5% in Ad-PTEN-GFP group, which significantly higher than that of 6.8% in Ad-GFP group_*P *< 0.01).

Cell cycle analysis by FCM showed that the percentage of G_2_/M phase cells in Ad-PTEN-GFP transfected K562 cells decreased, while the percentage of G_0_/G_1 _cells increased at each tested time point. Cell cycle was arrested at the G_0_/G_1 _phase (Figure 4 and Table 2).

**Table 2:** The comparison of Apoptosis rate and cell cycle change in K562 cells transfected with or without PTEN gene for different time.(%)

Group	Untransfected		Ad-GFP		Ad-PTEN-GFP	
	Apoptosis	G_2_/M	Apoptosis	G_2_/M	Apoptosis	G_2_/M
2d	0.2	30.2	0.8	29.3	1.5	28.1
5d	0.5	30.8	2.8	26.9	22.4	23.3
7d	1.1	29.7	4.1	32.9	30	13.6

### PTEN transfection and rapamycin treatment affected the growth and apoptosis of K562 cells

**Figure 5:**
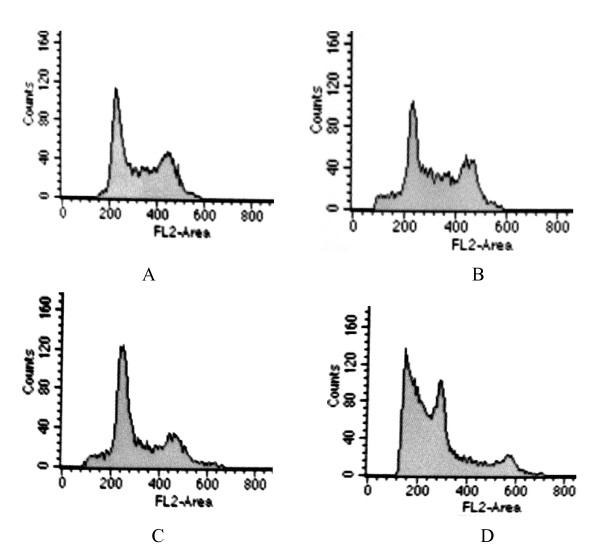
**The cell growth inhibition effect of rapamycin combined with PTEN transfection**.

Previous studies showed that rapamycin have anti-inflammatory, anti-tumor and immunosuppressive activities. Our results showed that the growth of K562 cells, which were transfected with PTEN and treated with RAPA (10 nmol/L) for 72 h, was significantly inhibited by 37.4%. This was significantly higher than that in K562 cells transfected with Ad-GFP and treated with 10 nmol/L RAPA (17.2%, *P *< 0.01) or that in K562 cells transfected with PTEN but without RAPA treatment (7.5%, *P *< 0.01). The apoptosis rate in K562 cells transfected with PTEN and treated with 10 nmol/L RAPA for 3 days was 31.5%, significantly higher than that in K562 cells transfected with Ad-GFP and treated with 10 nmol/L RAPA (6.8%, *P *< 0.01), or that in K562 cells transfected with Ad-PTEN-GFP but without RAPA treatment (6.1%, *P *< 0.01) (Fig 5).

### PTEN transfection and rapamycin treatment affected the expression of CyclinD1 and P27^kip1 ^mRNA

CyclinD1 and P27^kip1 ^mRNA and proteins were the key factors in modulating cell cycle. The present study showed that the expression level of CyclinD1 mRNA decreased in K562 cells transfected with Ad-PTEN-GFP and treated with 10 nmol/L RAPA for three days (0.121 ± 0.012), which was significantly lower than that in Ad-PTEN-GFP transfected K562 cells but without RAPA treatment (0.228 ± 0.036, *P *< 0.01), or that in Ad-GFP transfected and 10 nmol/L RAPA treated K562 cells (0.675 ± 0.049). The expression level of P27^kip1 ^mRNA was negatively correlated with that of CyclinD1 mRNA. P27^kip1 ^mRNA expressed at the top level (0.936 ± 0.061) in K562 cells transfected with Ad-PTEN-GFP and treated with 10 nmol/L RAPA for three days, which was significantly higher than that in K562 cells transfected with Ad-PTEN-GFP but without RAPA treatment (0.447 ± 0.032, *P *< 0.01), or that in Ad-GFP transfected K562 cells (0.060 ± 0.007, *P *< 0.01).

## Discussion

PTEN gene is a novel tumor suppressor gene. PTEN protein has protein phosphatase and lipid phosphatase dual activity, and has the activity against phosphatidylinositol 3, 4, 5- trisphosphate (PIP3), the major bioactive product of PI3-kinase. The reduction of PIP3 level suppresses the activation of Akt and allows apoptosis to occur. Homozygous PTEN deletion is embryonic lethal in mice and PTEN^+/- ^heterozygote mice may develop multiple tumors or immune system abnormalities. The inactivation of PTEN in leukemia can be induced by gene silencing, DNA methylation, transcriptional regulation or post-transcriptional modifications [14-16]. Our results showed that PTEN mNRA and protein expressed at very low level in K562 cells, a cell line established from a chronic myelogenous leukemia patient in blast crisis. The over-expression of wild-type PTEN protein after PTEN gene transfection showed an anti-proliferation and apoptosis induction effect in the K562 cells in a time-dependent manner, indicating that the wild type PTEN might have anti-leukemia effect. Although previous studies showed that a close correlation was found between the low expression of PTEN and the poor prognosis in some tumor patients including hematological malignancies [6,14,17,18] and the loss of PTEN gene disturbed the maintenance of quiescent HSCs and promoted leukemogenesis in mice [19], the exact mechanism of hypo-expression of PTEN in human leukemogenesis was not clear.

The mammalian target of rapamycin (mTOR) is a serine/threonine protein kinase, a downstream partner in PI3K/Akt pathway, which regulates protein translation, cell growth and apoptosis [20]. Abnormalities in the PI3K/AKT/mTOR signal pathway occurs in many solid or hematological malignancies. The present study also showed that mTOR mRNA and p-Akt protein expressed at high levels in K562 cells. After the cells were transfected with PTEN gene and treated with rapamycin, the expression levels of mTOR mRNA and p-Akt protein decreased accordingly. This result demonstrated that deregulated PI3K/AKT/mTOR signal pathway might contribute to the development of leukemia, and could be considered as a candidate target for the treatment of leukemia.

Rapamycin can stabilize the inhibitors of PI3K/AKT/mTOR signaling pathway and down-regulate the activity of mTOR. Studies showed that inhibiting mTOR with rapamycin decreased the phosphorylation of proteins in mTOR signaling pathway, induced cell cycle arrest at G_0_/G_1 _phase and distinctly rescued the differentiation of normal hematolpoietic stem cells (HSCs) and depleted leukemic stem cells. The regulation of HSCs and leukemic cells might be governed by cell-context-dependent, PTEN-mediated inhibition of mTOR [21]. The cell cycle arrest associated with the down-regulation of cyclinD1 and up-regulation of the cdk inhibitors p27^kip1 ^and p21^cip1 ^[9-11]. Therefore, inhibition of mTOR function represents a potential therapeutic strategy [10-13]. Our results showed that in the PTEN gene transfected K562 cells, over-expressed PTEN gene combined with the treatment of rapamycin significantly promoted the expression of P27^kip1 ^gene but inhibited the expression of the CyclinD1. Meanwhile, the percentage of cells in S and G_2_/M phase decreased and the cells in G_0_/G_1 _phase increased in the PTEN gene transfected K562 cells. These results suggested that PTEN gene and protein might inhibit the expression and the function of cyclins and cyclins-CDK but promote expression of P27^kip1 ^via down-regulating p-Akt and mTOR expression.

Several published papers showed that rapamycin played key role in inhibiting tumor proliferation by targeting mTOR and had synergistic anti-tumors effects with some chemotherapeutic drugs such as arsenic trioxide in several kinds of tumors [22]. The present study showed that PTEN gene transfection, together with the treatment of low concentration of rapamycin, had a higher growth inhibition effect and apoptosis induction effect in K562 cell, accompanied with the increased expression of PTEN mRNA and protein but decreased expression of mTOR, suggesting that rapamycin and PTEN might have synergic effect on inhibiting proliferation, promoting apoptosis and cell cycle arrest of leukemia cells via inhibiting the activity of PI3K/Akt/mTOR pathway. These results might provide a theoretical basis for the treatment of CML with new molecule targeting regimens such as PTEN transfection and the use of rapamycin.

## Competing interests

The authors declare that they have no competing interests.

## Authors' contributions

SYW and HC participated in the measurement of the target proteins by western blotting. Cell cycle distribution assay was carried out by ZYN and SHL. ZYC, XYY carried out cell culture, transfection, measurement of growth inhibition of rapamycin, statistics and writing manuscript. LP, XLG, ZYC, XYY conceived of the study, and participated in its design and coordination. LP revised the manuscript finally. All of us showed our opinion after plenty of reading. All authors read and approved the final manuscript.
